# 5-aza-2′-deoxycitidine inhibits cell proliferation, extracellular matrix formation and Wnt/β-catenin pathway in human uterine leiomyomas

**DOI:** 10.1186/s12958-021-00790-5

**Published:** 2021-07-08

**Authors:** María Cristina Carbajo-García, Ana Corachán, Marina Segura-Benitez, Javier Monleón, Julia Escrig, Amparo Faus, Antonio Pellicer, Irene Cervelló, Hortensia Ferrero

**Affiliations:** 1 Fundación IVI, Instituto de Investigación Sanitaria La Fe, Avenida Fernando Abril Martorell 106, Torre A, Planta 1ª, 46026 Valencia, Spain; 2 Departamento de Pediatría, Obstetricia y Ginecología, Universidad de Valencia, Valencia, Spain; 3 Hospital Universitario y Politécnico La Fe, Valencia, Spain; 4 IVIRMA Rome, Rome, Italy

**Keywords:** Uterine leiomyoma, Epigenetics, 5-aza-2′-deoxycitidine, Cell proliferation, Wnt/β-catenin pathway

## Abstract

**Background:**

Uterine leiomyoma is a benign tumor with unclear pathogenesis and inaccurate treatment. This tumor exhibits altered DNA methylation related to disease progression. DNMT inhibitors as 5-aza-2′-deoxycytidine (5-aza-CdR), have been suggested to treat tumors in which DNA methylation is altered. We aimed to evaluate whether DNA methylation reversion with 5-aza-CdR reduces cell proliferation and extracellular matrix (ECM) formation in uterine leiomyoma cells to provide a potential treatment option.

**Methods:**

Prospective study using uterine leiomyoma and adjacent myometrium tissues and human uterine leiomyoma primary (HULP) cells (*n* = 16). In tissues, gene expression was analyzed by qRT-PCR and DNMT activity by ELISA. Effects of 5-aza-CdR treatment on HULP cells were assessed by CellTiter, western blot, and qRT-PCR.

**Results:**

DNMT1 gene expression was higher in uterine leiomyoma vs myometrium. Similarly, DNMT activity was greater in uterine leiomyoma and HULP cells (6.5 vs 3.8 OD/h/mg; 211.3 vs 63.7 OD/h/mg, respectively). After 5-aza-CdR treatment on HULP cells, cell viability was reduced, significantly so at 10 μM (85.3%). Treatment with 10 μM 5-aza-CdR on HULP cells significantly decreased expression of proliferation marker PCNA (FC = 0.695) and of ECM proteins (COLLAGEN I FC = 0.654; PAI-1, FC = 0.654; FIBRONECTIN FC = 0.733). 5-aza-CdR treatment also decreased expression of Wnt/β-catenin pathway final targets, including WISP1 protein expression (10 μM, FC = 0.699), *c-MYC* gene expression (2 μM, FC = 0.745 and 10 μM, FC = 0.728), and *MMP7* gene expression (5 μM, FC = 0.520 and 10 μM, FC = 0.577).

**Conclusions:**

5-aza-CdR treatment inhibits cell proliferation, ECM formation, and Wnt/β-catenin signaling pathway targets in HULP cells, suggesting that DNA methylation inhibition is a viable therapeutic target in uterine leiomyoma.

**Supplementary Information:**

The online version contains supplementary material available at 10.1186/s12958-021-00790-5.

## Background

Uterine leiomyomas (UL) are monoclonal benign tumors originating from smooth muscle cells located in the myometrium (MM) that affect to 25–30% of women of reproductive age [1, 2]. Prevalence of uterine leiomyomas in African American women is three times higher than Caucasian women [3, 4]. Around 30% of patients with leiomyomas present symptoms such as excessive uterine bleeding, anemia, pelvic pain, infertility, recurrent pregnancy loss, and/or preterm birth [5]. Although the gold-standard treatment for leiomyoma is surgical myomectomy or hysterectomy, other less invasive hormonal treatments, such as gonadotropin releasing-hormone agonist (aGnRH) [6] or ulipristal acetate (UPA) have been used to treat leiomyomas [7]. However, these treatments present side effects such as menopausal symptoms or hepatic damage [8], and once the treatment is stopped, leiomyomas enlarge again, in most cases recovering their initial size after 6 months [9]. For this reason, there is not an effective therapy to treat uterine leiomyoma with minimal side effects, despite being the most prevalent gynecological tumor.

Although pathogenesis of leiomyomas remains unclear, many factors have been proposed to contribute in the development of uterine leiomyomas. These factors include steroid hormones such as estrogens [10] and progesterone [11]; growth factors such as tumor growth factor beta (TGF-β), vascular endothelial growth factor (VEGF), acidic fibroblast growth factor (FGF), and basic FGF (bFGF) [12]; alterations of the Wnt/β-catenin pathway [13]; and genetic and epigenetic alterations [14]. Different subtypes of genetic mutations are described as a possible cause of leiomyoma development, including high mobility group AT-hook 2 (*HMGA2*) rearrangements, mediator complex subunit 12 (*MED12*) mutations, biallelic inactivation of fumarate hydratase (*FH*), and deletions affecting collagen type IV alpha 5 and alpha 6 (*COL4A5* and *COL4A6*) [15]. In addition, some environmental factors increase risk for developing uterine leiomyomas; these include high body mass index (BMI), meat consumption, alcohol consumption, hypertension, and vaginal infections [16–18]. These data suggest that epigenetic factors are also involved in the development of uterine leiomyoma. While genetic mutations are difficult to reverse, some epigenetic changes can be reversed, for example by chemical agents. This possibility suggests that there could be new therapeutic options for patients with UL.

DNA methylation is a widely studied epigenetic mark that consists of the addition of a methyl group to the 59-carbon of the cytosine ring through a covalent union within the CpG island after replication [19]. Specifically, methylation of a promoter CpG island triggers binding of proteins that condense the chromatin and make the promoter region inaccessible to transcription factors, blocking transcription initiation and, thereby, repressing gene expression [20]. DNA methylation is catalyzed by DNA methyltransferases (DNMT), of which there are three main types: DNMT1, DNMT3A, and DNMT3B [14]. DNMT1 maintains DNA methylation patterns during DNA replication; in contrast, DNMT3A and DNMT3B establish new methylation patterns and are therefore called de novo methyltransferases [21]. Aberrant DNA methylation, through oncogene hypo-methylation and tumor suppressor gene hyper-methylation, is involved in tumorigenesis of various cancer types, such as prostate cancer or neuroblastoma [22–25]. In UL, some studies reported differential expression of DNMTs and an aberrant DNA methylation compared with the adjacent MM, along with repression of tumor suppressor genes and other genes involved in cell cycle regulation, cell growth, migration, and extracellular matrix (ECM) formation [26–30]. Of particular interest is the Wnt/β-catenin pathway, which regulates proliferation, survival, migration and differentiation of many cell types and is dysregulated in numerous cancers [31–34] and uterine leiomyomas cells [7, 13, 35–37]. There is potential that aberrant DNA methylation affects this pathway in UL as it does in other tumor types, such as colorectal cancer [38]. For other tumors with altered DNA methylation that is related to disease progression, the use of DNMT inhibitors such as 5-aza-2′-deoxycytidine (5-aza-CdR) can offer effective treatment. 5-aza-CdR is a nucleoside analog that acts as a DNA demethylating agent through inhibition of DNMTs (DNMT1/3A/3B) [19], leading to changes in gene reactivation. 5-aza-CdR has been demonstrated that induce cell cycle arrest, inhibition of cell differentiation, and cell death by inhibiting post-replication methylation of DNA in human and mouse endometrium [39, 40]. Given the altered DNA methylation patterns and DNMT expression in UL, demethylation of human uterine leiomyoma cells by this DNMT inhibitor could reduce leiomyoma size, offering a new therapeutic option. However, the effect of 5-aza-CdR on UL cells is not well studied; further research is necessary to determine how this inhibitor affects the regulation of molecular mechanisms involved in leiomyoma development.

Here, we evaluated the effect of 5-aza-CdR in human uterine leiomyoma primary (HULP) cells on cell proliferation, apoptosis, ECM formation, and the Wnt/β-catenin pathway to test its potential as a new therapeutic option for leiomyoma.

## Methods

### Human tissue collection

Human UL and adjacent MM were collected from premenopausal women aged 31–48 years undergoing myomectomy or hysterectomy due to symptomatic UL pathologies (*n* = 16) without any previous hormonal treatment for the last 3 months (Supplemental Table 1).

### Ethical approval

This study was approved by Clinical Ethics Committee at Hospital Univeristario y Politecnico La Fe (Spain) (2018/0097), and all participants provided informed consent.

### Human uterine leiomyoma primary cell isolation

UL and MM fragments were mechanically dissected into small pieces that were incubated at 37 °C with 2 mg/mL type II collagenase (Labclinics, Spain) and 1 mg/mL DNase I (Sigma-Aldrich, Saint Louis, MO) to obtain single-cell suspensions. Subsequently, cells were filtered through 50-μm polyethylene filters (Partec, Celltrics) to remove cellular clumps and undigested tissue. Isolated cells were incubated in vitro at 37 °C and 5% CO_2_ in culture medium [Dulbecco’s Modified Eagle Medium (DMEM)/F-12 (Gibco, Waltham, MA) with 10% fetal bovine serum (FBS) and antibiotic-antimycotic solution] for subsequent experiments of DNMT activity and 5-aza-CdR treatment.

### Study of DNMT activity in vivo and in vitro

To evaluate DNMT activity over time in HULP and MM cells isolated from UL and adjacent MM tissue (*n* = 3), cells were incubated in culture medium for 7, 9, or 10 days. Subsequently, HULP and MM cells were collected, and nuclear protein extracts were isolated using EpiQuik Nuclear Extraction Kit (Epigentek, Brooklyn, NY), which was also used to obtain nuclear proteins from UL and MM tissues (*n* = 7). DNMT activity in tissues and in vitro cultured HULP and MM cells was measured with EpiQuik DNMT Activity/Inhibition ELISA Easy Kit immunoassay (Epigentek, Brooklyn, NY) using a microplate reader (Synergy HT, Bio-Tek). The ratio of methylated DNA was measured at a wavelength of 450 nm. The activity of DNMT enzymes, which is proportional to the optical density (OD) intensity measured, was calculated according to the formula provided by the manufacturer:

$$ DNMT\kern0.34em Activity\kern0.28em \left( OD/h/ mg\right)=\frac{\left( Sample\kern0.28em OD- Blank\kern0.28em OD\right)}{\left( Protein\kern0.34em amount\kern0.28em \left(\mu g\right)\times hour\right)}\times 100 $$

### 5-aza-2′-deoxycitidine treatment

To determine the effect of 5-aza-CdR on HULP cells, cells were incubated in culture medium (day 0 = D_0_) until achieving a confluence of 70% (day 3 = D_3_). Then, they were starved in serum-free medium overnight and treated (day 4 = D_4_) with different doses of 5-aza-dC (Abcam, Cambridge, UK) (*n* = 8): 0 μM (control), 2 μM (low-dose), 5 μM 5-aza-CdR (middle-dose), 10 μM (high-dose) for 72 h (day 7 = D_7_). Medium was changed every 24 h.

### Cell viability assay

HUPL cells (*n* = 16) were cultured in 96-well plate in culture medium; once 70% of confluence was achieved, cells were starved in serum-free medium overnight and treated (D_4_) with 0, 2, 5, or 10 μM of the DNMT inhibitor 5-aza-CdR for 72 h (D_7_). Medium was changed every 24 h. After this period, the quantity of viable cells in proliferation was measured with CellTiter 96 AQueous One Solution Cell Proliferation Assay (Promega, Madison, WI) and absorbance was measured on a microplate reader (Synergy HT, Bio-Tek) at 490 nm.

### Protein extraction and western blot analysis

Proteins from HULP cells (*n* = 8) treated with/without 5-aza-CdR were extracted using radioimmunoprecipitation assay (RIPA) buffer containing protease inhibitors. Subsequently, HULP cells lysates (10 μg to 20 μg of protein) were analyzed by 12% sodium dodecyl sulphate-polyacrylamide gel electrophoresis (SDS-PAGE) and then transferred to PVDF membranes. Western blot (WB) analysis was conducted to measure expression of ECM protein Plasminogen activator inhibitor-1 (PAI-I; sc-5297) (1:200), proliferating cell nuclear antigen (PCNA; sc-56) (1:200), Wnt1-inducible-signaling pathway protein 1 (WISP1; sc-133,126) (1:200), B-cell lymphoma-2 (BCL2; sc-7382) (1:200), and BCL2 associated-X (BAX; sc-20,067) (1:200) from Santa Cruz Biotechnology (Santa Cruz, CA). Expression of extracellular matrix proteins COLLAGEN I (COL-I;70R-CR007X) (1:1000, Fitzgerald, Acton, MA) and FIBRONECTIN (F3648) (1:2000, Sigma-Aldrich) was also evaluated. Antigen-antibody complex was detected with SuperSignal West Femto Maximun Sensitivity Substrate (ThermoFisher, Waltham, MA) and specific protein bands were visualized by chemiluminescence imaging using the LAS-3000 Imaging-System (Fujifilm, Tokyo, Japan). The intensity of each protein band was quantified with ImageJ software (National Institutes of Health, Bethesda, MD) and normalized in relation to its corresponding housekeeping protein, β-actin (1:1000; sc-47,778).

### Gene expression analysis: DNMT and Wnt/β-catenin pathway

Total RNA from UL and MM tissues was extracted with TRIzol reagent (Fisher Scientific, Waltham, MA) for the study of *DNMT1* expression. Total RNA from HULP cells treated with/without 5-aza-CdR was obtained using RNeasy Mini kit (Qiagen, Hilden, Germany) to assess expression of final targets in the Wnt/B-catenin pathway. cDNA was synthesized using PrimeScript RT reagent Kit (Takara, Japan). Quantitative real-time polymerase chain reaction (qRT-PCR) was performed with StepOnePlus System (Applied Biosystems, Foster City, CA) using PowerUp SYBR Green (Fisher Scientific, Waltham, MA). Gene expression of final targets of Wnt pathway, MYC proto-oncogene *(c-MYC)* and Matrix Metallopeptidase 7 (*MMP7),* was analyzed in HULP cells treated with and without 5-aza-CdR (*n* = 8). In addition, *DNMT1* expression was analyzed in UL and MM tissues (*n* = 8). Data were normalized with Glyceraldehyde-3-Phosphate Dehydrogenase (*GAPDH),* a housekeeping gene with minor variability. Primers were designed using Primer Quest Tool (DNA Integrated Technologies, Coralville,IA) (Supplemental Table II). ΔΔCt method was used to calculate fold change.

### Statistical analysis

GraphPad Prism 8.0 was used for statistical analyses and graphic generation (San Diego, CA). Normality and logarithmic tests (Anderson-Darling, D’Agostino & Pearson, Shapiro-Wilk and Kolmogorov-Smirnov test) were performed to analyze the distribution of our results. A paired t-test was performed for DNMT activity study in UL vs MM tissue as well as HULP and MM cells. The Wilcoxon test for paired samples was performed to analyze qRT-PCR results of DNMT1 gene expression in UL tissue and adjacent MM. Repeated measures one-way ANOVA test with the Geisser-Greenhouse correction was performed for qRT-PCR analysis of multicomponent variants of *c-MYC* and *MMP7;* for WB analysis of WISP1, PCNA, and COL-I; and for cell viability assay of HULP and MM cells. Friedmann test was used to analyze BAX-BCL2 ratio, PAI-I, and FIBRONECTIN. Results of 5-aza-CdR treatment were normalized and compared with control HULP cells without treatment. Data are presented as mean ± standard deviation (SD). *p*-value < 0.05 was considered statistically significant.

## Results

### DNTM1 gene expression and DNMT activity in UL vs MM tissues

**Fig. 1:**
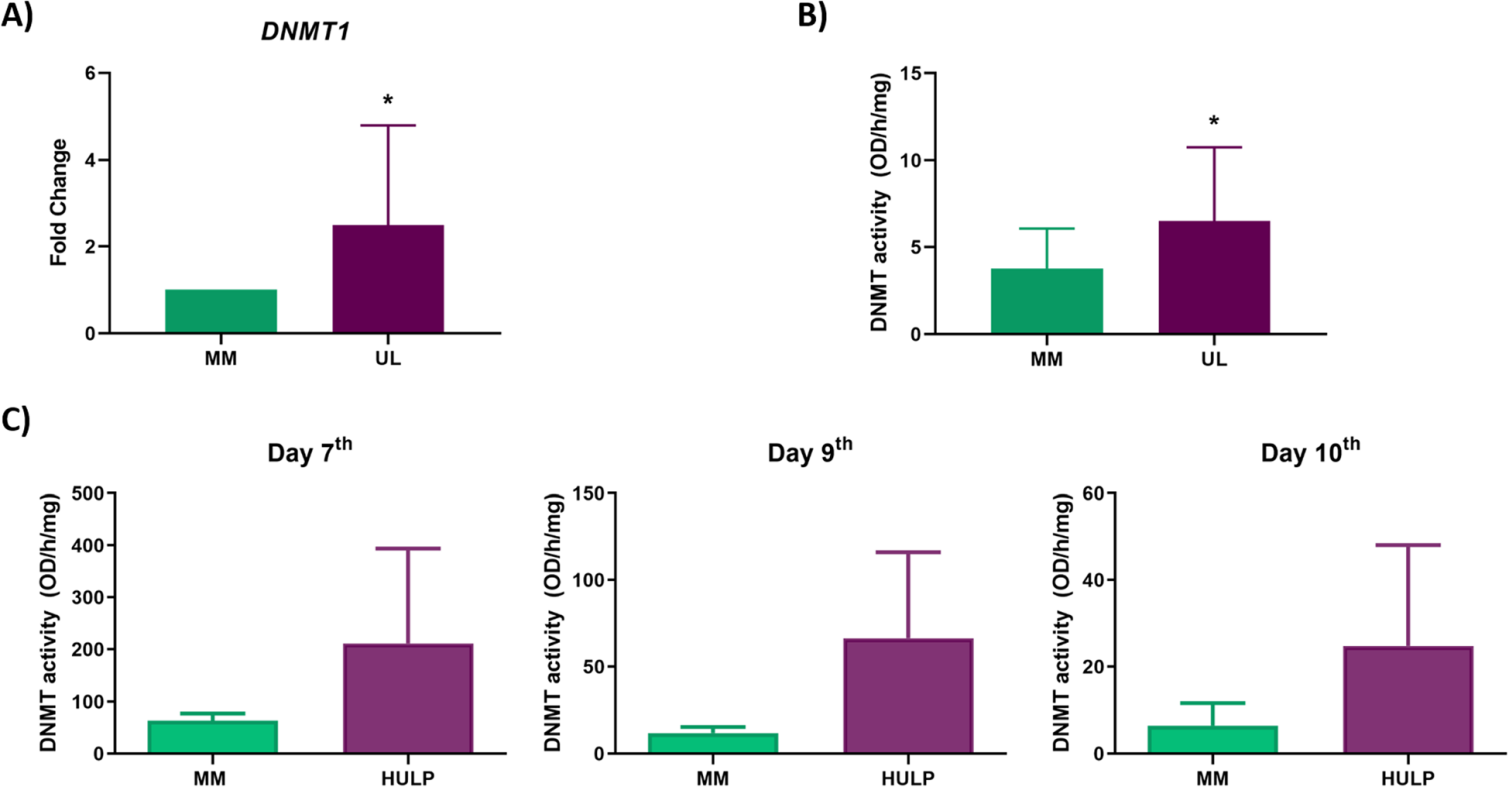
DNMT status in UL compared with adjacent MM. **A** Gene expression levels of *DNMT1* in UL compared to MM tissues (*n* = 14) represented as fold change. **B** DNMT activity (OD/h/mg) of UL compared to MM tissues (*n* = 7). **C** Effects of cell culture on DNA methyltransferase (DNMT) activity of HULP and MM cells (*n* = 3) cultured for 7 days, 9 days, and 10 days. Data are expressed as the mean value ± standard error (SEM). * p-value < 0.05

To examine whether increased global DNA methylation previously observed in UL vs MM was due to a higher expression of DNMT enzymes, *DNMT1* gene expression was analyzed by qRT-PCR in UL and MM tissues. UL tissue showed a statistically significant higher gene expression of *DNMT1* compared to MM tissue (fold change = 2.49, *p*-value = 0.0295) (Fig. 1A). Furthermore*,* DNMT activity was measured by ELISA in UL vs MM tissues to determine the correlation between gene expression and DNMT activity. Significantly higher DNMT activity was observed in UL tissue compared to adjacent MM tissue (6.50 vs 3.76 OD/h/mg, *p*-value = 0.026) (Fig. 1B).

### DNMT activity in HULP and myometrial cells in vitro

Since epigenetic changes depend on the environment, experiments were conducted to determine the correlation between DNMT activity in vivo in UL and adjacent MM tissue and HULP and MM cells cultured in vitro. For this purpose, DNMT activity of HULP and MM cells was measured at 7, 9, and 10 days of in vitro culture. DNMT activity was greater in HULP cell compared to MM cells at day 7 (211.30 vs 63.67 OD/h/mg, *p*-value = 0.284), day 9 (66.41 vs 11.88 OD/h/mg, *p*-value = 0.217), and day 10 (24.75 vs 6.47 OD/h/mg, *p*-value = 0.337) (Fig. 1C). These results corroborated that the increased DNMT activity observed in UL compared to MM tissues is not modified in vitro by cell culture conditions (Fig. 1C).

### In vitro effects of 5-aza-2′-deoxycytidine on cell proliferation in HULP cells

**Fig. 2:**
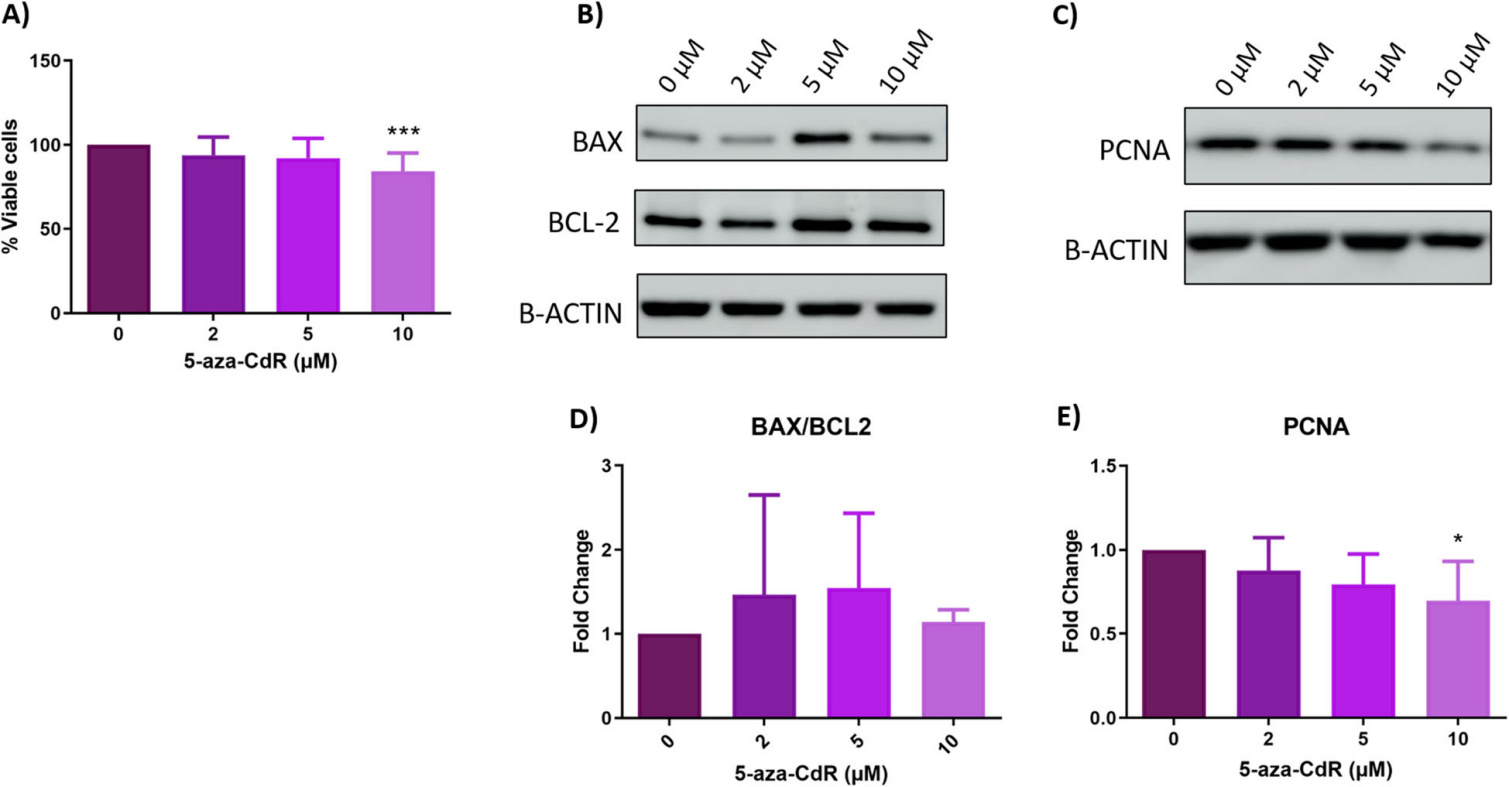
Effect of 5-aza-CdR treatment on cell survival in HULP cells. **A** Percentage of viable HULP (*n* = 16) cells after 72 h of treatment with 0, 2, 5, or 10 μM of 5-aza-CdR. **B** Representative images of protein expression levels for BCL2 (23 kDa) and BAX (26 kDa) and **C** PCNA (36 kDa) analyzed in HULP (*n* = 8) after treatment with 5-aza-CdR at 0, 2, 5, or 10 μM (D-E) Means and standard deviations of normalized data of BAX/BCL2 ratio and PCNA, both represented as fold-change. * p-value < 0.05 *** p-value < 0.001

To demonstrate the antiproliferative effect of 5-aza-CdR on HULP cells, we measured the number of proliferating cells. Treatment with 5-aza-CdR decreased the percentage of viable HULP cells in a dose-dependent manner, being this decrease statistically significant with high-dose (10 μM) treatment in HULP cells (93.93% at 2 μM, *p*-value = 0.114; 92.15% at 5 μM, *p*-value = 0.053; 85.25% at 10 μM, *p*-value = 0.0001) compared to 0 μM (Fig. 2A).

Subsequently, to clarify whether this decrease in viable cells was due to cell death increase and/or to cell proliferation decrease, protein markers of apoptosis status and proliferation were analyzed in 5-aza-CdR-treated HULP cells by western blot. We measured BAX (proapoptotic) and BCL2 (anti-apoptotic) protein expression (Fig. 2B) and did not observe an increased apoptosis (no BCL2 upregulation or BAX downregulation). Moreover, we calculated the BAX/BCL2 ratio to determine the susceptibility to apoptosis in 5-aza-CdR-treated HULP cells compared with HULP cells without treatment (0 μM). Although the BAX/BCL2 ratio showed a trend to increase in HULP cells after treatment, no significant differences were observed with 2, 5, or 10 μM of 5-aza-CdR [fold change (FC) = 1.46, *p*-value = 0.099; FC = 1.54, *p*-value = 0.158; FC = 1.13, *p*-value = 0.099, respectively) compared to untreated cells (Fig. 2D). Therefore, 5-aza-CdR-treated HULP cells did not show a significant change in apoptosis. To further analyze the decrease in cell viability observed in 5-aza-CdR-treated HULP cells, we assessed effect of 5-aza-CdR on cell proliferation by analyzing protein expression of the gold-standard proliferation marker PCNA using western blot in 5-aza-CdR-treated and untreated HULP cells (Fig. 2C). 5-aza-CdR treatment decreased PCNA protein expression in HULP cells in a dose-dependent manner, reaching statistical significance at the high dose (2 μM FC = 0.877, *p*-value = 0.099; 5 μM FC = 0.794, *p*-value = 0.055; 10 μM FC = 0.695, *p*-value = 0.034, respectively) (Fig. 2E).

### In vitro effects of 5-aza-2′-deoxycytidine on extracellular matrix in HULP cells

**Fig. 3:**
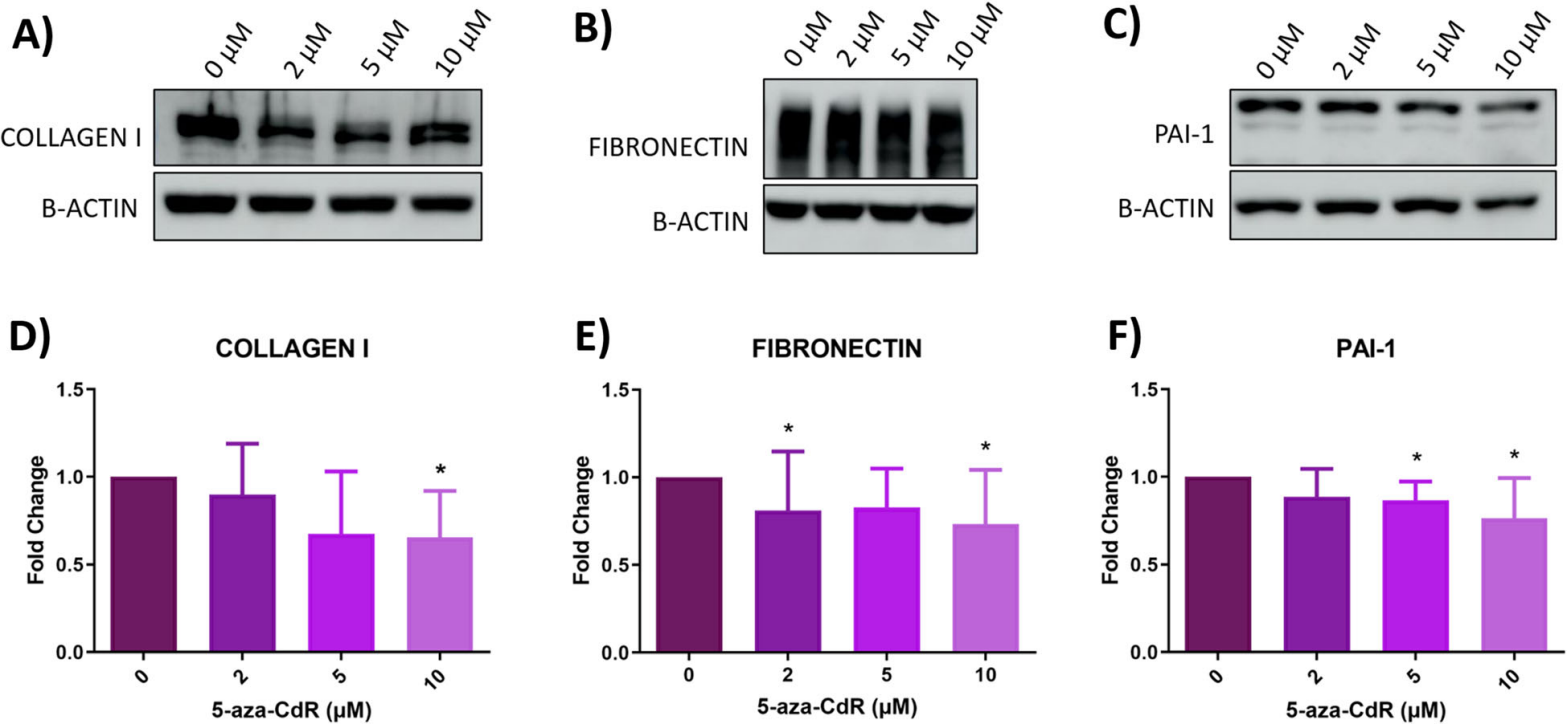
Extracellular matrix evaluation in HULP cells after 5-aza-CdR treatment. Representative images of protein expression levels for **A** COLLAGEN I (140 kDa), **B** FIBRONECTIN (220 kDa), and **C** PAI-1 (50 kDa) and normalized quantitative protein expression of **D** COLLAGEN I, **E** FIBRONECTIN, and **F** PAI-1 in the HULP cells at different treatment groups with 5-aza-CdR at 0, 2, 5, or 10 μM for 72 h (*n* = 8). Data are represented as mean and deviations of fold change. * p-value < 0.05

To evaluate ECM status in HULP cells after 5-aza-CdR treatment, expression of proteins involved in ECM formation, such as COLLAGEN I, FIBRONECTIN, and PAI-1, was determined by western blot (Fig. 3A, B, and C, respectively). 5-aza-CdR treatment decreased COLLAGEN I expression in a dose-dependent manner, reaching statistical significance at 10 μM (FC = 0.654, *p*-value = 0.023; Fig. 3D). In addition, a tend to decrease COLLAGEN I was observed at 2 μM and 5 μM (FC = 0.6540.897, *p*-value = 0.729; FC = 0.675, *p*-value = 0.104, respectively) (Fig. 3D). Although no changes were found in FIBRONECTIN expression at 5 μM (FC = 0.826, *p*-value = 0.244), a statistically significant reduction was observed at 2 μM (FC = 0.812, *p*-value = 0.020) and 10 μM (FC = 0.733, *p*-value = 0.035) (Fig. 3E). Finally, PAI-1 expression was significantly decreased compared to control group (0 μM) after 5-aza-CdR treatment at 5 μM and 10 μM (FC = 0.865, *p*-value = 0.035; FC = 0.766, *p*-value = 0.020, respectively) (Fig. 3F). No statistically significant differences were found at 2 μM 5-aza-CdR (FC = 0.885, *p*-value = 0.244). These results showed that 5-aza-CdR inhibited ECM protein expression in HULP cells in vitro in a dose-dependent manner.

### In vitro effects of 5-aza-2′-deoxycytidine on Wnt/β-catenin signaling pathway in HULP cells

**Fig. 4:**
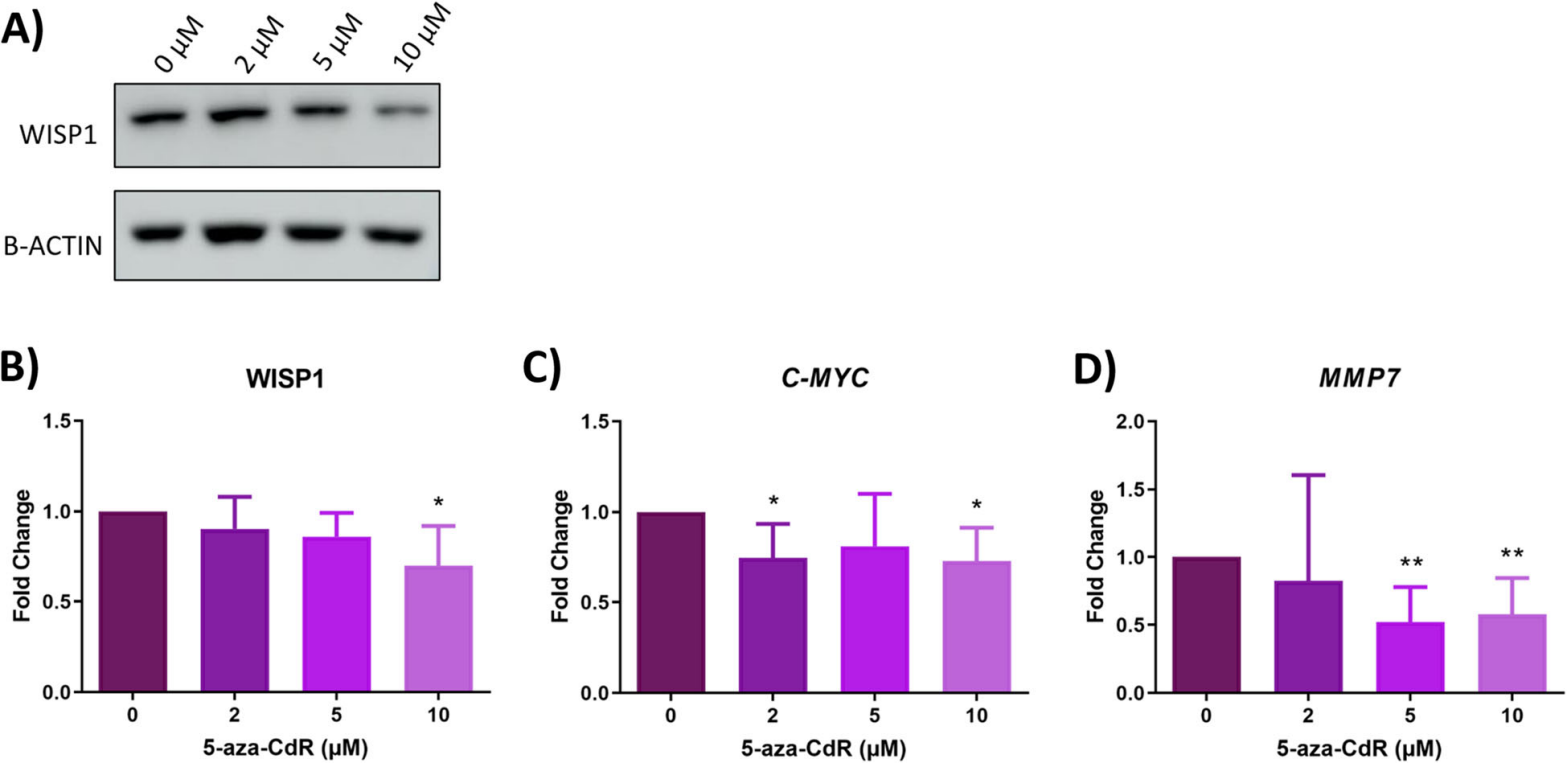
Wnt/β-catenin signaling pathway analysis in HULP cells after DNMT inhibition with 5-aza-CdR treatment. **A** Representative images of protein expression for WISP1 in HULP cells after treatment with 5-aza-CdR at 0, 2, 5, or 10 μM for 72 h. **B** Normalized quantitative protein expression of WISP1 represented as mean and deviations of fold-change. Gene expression levels of **C** *c-MYC* and **D** *MMP7* in HULP cells treated for 72 h with 2, 5, or 10 μM of 5-aza-CdR compared to untreated HULP cells (0 μM), represented as fold-change. * p-value < 0.05 ** p-value < 0.01

To assess the effect of 5-aza-CdR treatment on Wnt/β-catenin pathway on HULP cells, we measured the protein expression of WISP1, a Wnt/β-catenin target protein, in HULP cells in presence and absence of 5-aza-CdR treatment by western blot. Results revealed an inhibition of WISP1 protein expression in HULP cells treated with 5-aza-CdR compared to control group (0 μM) in a dose-dependent manner treatment (Fig. 4A), reaching statistical significance at 10 μM (2 μM FC = 0.903, *p*-value = 0.408; 5 μM FC = 0.860, *p*-value = 0.071; 10 μM FC = 0.699, *p*-value = 0.026, respectively) (Fig. 4B).

To further analyze the regulation of Wnt/β-catenin signaling pathway by 5-aza-CdR treatment in HULP cells, gene expression levels of *c-MYC* and *MMP7*, as final targets of Wnt/β-catenin pathway, were determined in these cells by qRT-PCR. *c-MYC* was significantly downregulated in HULP cells after 5-aza-CdR treatment at all doses tested compared to HULP cells without treatment, reaching significance at 2 μM and 10 μM doses (2 μM FC = 0.745, *p*-value = 0.028; 10 μM FC = 0.728, *p*-value = 0.019, respectively) (Fig. 4C), while no significant differences were found at 5 μM (FC = 0.810, *p*-value = 0.287). Finally, a significantly decrease of *MMP7* expression was observed in HULP cells after 5-aza-CdR treatment with 5 μM and 10 μM compared to HULP without treatment (5 μM FC = 0.520, *p*-value = 0.003, 10 μM FC = 0.577, *p*-value = 0.007, respectively) (Fig. 4D). No significant differences were found in *MMP7* expression at 2 μM (FC = 0.824, *p*-value = 0.860). Thus, 5-aza-CdR inhibited final targets of Wnt/β-catenin pathway in HULP cells in vitro in a dose-dependent manner.

## Discussion

Our study shows that 5-aza-2′-deoxycitidine inhibits cell proliferation, ECM formation, and Wnt/β-catenin pathway in human uterine leiomyoma primary cells, suggesting DNA methyltransferases inhibitors as a new effective option for uterine leiomyoma treatment. UL is a multifactorial disease with an unclear pathogenesis and ineffective treatment [6–9]. For this reason, determining the molecular mechanisms involved in UL growth is necessary to better understand its pathogenesis, as well as define molecular targets to develop new therapeutic options against them. Factors contributing to UL pathogenesis include genetic mutation, epigenetic modifications, and several growth factors [14]. In addition, estrogen and progesterone are recognized as promoters of UL growth [14]. Accordingly, a significant growth of UL during the first trimester of pregnancy is associated with hormonal changes [41]. In this regard, epigenetic modifications could play an important role in steroid hormonal “cross-talk” for UL development and growth [14, 42]. Epigenetics is emerging as a new hallmark of tumor development with a high therapeutic application because of its potential for reversal [23, 25]. In fact, abnormal DNA methylation is found in UL compared to MM tissue, where tumor suppressor genes are hypermethylated, which contributes to the development of this tumor [14, 21, 27–30]. Based on these findings, we focused our study on DNMT methylation reversion by DNMT inhibitor 5-aza-2′-deoxycitidine as new therapeutic option for UL.

For this purpose, we first studied DNMT expression and activity of UL and MM tissue and we corroborated that there is a higher *DNMT1* expression and DNMT activity in UL compared to MM tissue. Interestingly, similar findings were reported by others, who associated the aberrant DNA methylation found in UL with an increased DNMT1 and DNMT3a mRNA expression in tumor samples compared to myometrium [26, 27]. Based on these finding, the use of DNMT inhibitors to reverse the aberrant DNA methylation found in UL could be a good therapeutic approach to treat them. Secondly, since epigenetic changes depend on the environment, we studied DNMT expression and activity in cell culture to confirm that an in vitro model maintains the epigenetic feature from origin tissue. We proved for the first time that the increased DNMT activity observed in UL tissue is maintained under cell culture conditions over time in HULP compared to MM cells, confirming the reproducibility of the in vitro model. This approach allows the study of epigenetic modifications such as DNMT methylation reversion using DNMT inhibitor.

In this regard, the use of DNMT inhibitor 5-aza-CdR is widely described in several tumor types, such as colorectal, bladder, and pancreatic cancer, demonstrating antiproliferative effects [38, 43, 44]. Uncontrolled proliferation is one of the hallmarks of cancer, and tumorigenic cells present greater proliferation and lesser apoptosis than normal cells. Accordingly, several authors showed that UL growth is due to an increased proliferation of UL cells [7, 13]. Since UL is associated with aberrant DNA methylation and increased cell proliferation, reversion of DNMT methylation by 5-aza-CdR could be an efficient treatment to reduce UL size due to its antiproliferative effects. Here, we found that 5-aza-CdR treatment of HULP cells inhibited the number of proliferating cells, and this decrease was due to a reduction in cell proliferation, as demonstrated by decreased PCNA protein expression. These findings demonstrated the antiproliferative effect in UL previously described in different tumors [38, 43, 44]. Meanwhile, apoptosis did not increase after 5-aza-CdR treatment, suggesting that this treatment would not be toxic for HULP cells. Based on our results, 5-aza-CdR would decrease cell proliferation in HULP cells, highlighting its potential as a therapeutic option to reduce UL growth.

UL growth is due not only to cell proliferation, but also to an excessive synthesis and deposition of ECM. Thus, evaluating the effect of DNMT inhibitors on ECM protein expression is necessary to assess the possible use of these inhibitors as a new therapeutic option for UL. Our results showed that 5-aza-CdR treatment significantly decreased the expression of ECM-associated proteins such as FIBRONECTIN, COLLAGEN I, and PAI-1. These findings suggested an important role of 5-aza-CdR in the regulation of key fibrotic proteins involved in UL expansion. In line with this, other studies demonstrated the success of treatments targeting ECM formation as possible therapeutic option to reduce UL grown, such as Vitamin D treatment in the Eker rat model [45], in a xenograft mouse model [46], and in human leiomyoma cells [47]. Hence, 5-aza-CdR reduced ECM expansion and cell proliferation in HULP cells, which counteracts UL growth, offering a promising candidate to treat this tumor.

Wnt/β-catenin pathway is involved in several cellular functions, being a key regulator of cell proliferation and ECM production. This pathway is dysregulated in several cancers [31–34, 48] and UL [7, 13, 35]. This pathway’s implications in colorectal cancer (CRC) are well studied [49]. In CRC, the effect of 5-aza-CdR in reduction of cell self-renewal through Wnt/β-catenin pathway inhibition is demonstrated, showing the important role of epigenetic modification on Wnt/β-catenin pathway regulation and its implication in cancer development [38]. In UL, previous studies suggested that targeting Wnt/β-catenin pathway can be a promising therapeutic approach because of their aberrant activation in UL cells compared to MM cells [13, 36, 37, 50]. Due to the increased activation of Wnt/β-catenin pathway and aberrant DNA methylation found in UL, we analyzed if DNA methylation reversion by 5-aza-CdR treatment had an effect on the Wnt/β-catenin pathway in HULP cells. We assessed protein or mRNA expression of final targets of the Wnt/β-catenin pathway, such as WISP1, *c-MYC,* and *MMP7,* because of their tight association with tumor progression. We found that both *c-MYC* gene expression and WISP1 protein expression were downregulated in treated HULP cells. *c-MYC* is one of the most studied final targets of the Wnt/β-catenin pathway. It is a protooncogene involved in cell cycle progression [51] whose deregulation has been linked to an aberrant Wnt/β-catenin pathway expression in CRC [52]. WISP1 has been identified as an oncogene in several cancer types such as glioblastoma [53], CRC [54], and colon cancer [55], with involvement in tumor proliferation, migration, and poor prognosis. Inhibition of WISP1 decreases cell proliferation and invasion through increasing apoptosis and blocking cell cycle in glioblastoma and colon cancer cells [53, 55]. Therefore, the reduction of WISP1 and *c-MYC* expression observed in our study suggest that DNMT inhibition by 5-aza-CdR could impede cell proliferation via Wnt/β-catenin pathway inhibition. Finally, *MMP7* is a metalloprotease involved in ECM physiology, epithelial-mesenchymal transition, and tumor invasion that is overexpressed in some cancer types [56]. In this regard, a study in hepatocellular carcinomas demonstrated the inhibition of cell migration and invasion after diminishing MMP7 [57]. Accordingly, we observed a decrease in *MMP7* gene expression after 5-aza-CdR treatment in HULP cells, suggesting a lower invasive capacity of HULP-treated cells via Wnt/β-catenin pathway inhibition.

Based on these findings, we suggest that DNA methylation is involved in Wnt/β-catenin pathway regulation and, consequently, in cell proliferation and ECM formation in HULP cells, proposing 5-aza-CdR as a treatment to reduce uterine leiomyoma growth. Further studies are necessary to determine the role of DNA methylation on Wnt/β-catenin pathway, as well as the in vivo effect of 5-aza-CdR on UL.

## Conclusions

Our study demonstrated for the first time that 5-aza-CdR reduces cell proliferation, ECM formation, and Wnt/β-catenin pathway in HULP cells in vitro*.* Yet, apoptosis appears unaffected. Based on our findings, we suggest DNMT inhibitors such as 5-aza-CdR could offer a new therapeutic option to treat patients with uterine leiomyoma.

## Supplementary Information

**Additional file 1.**

**Additional file 2.**

## Data Availability

All data generated or analyzed during this study are included in this published article [and its supplementary information files].

## Abbreviations

5-aza-CdR: 5-aza-2′-deoxycytidine.
AGnRH: Gonadotropin releasing-hormone agonist
BAX: BCL2 associated-X
BCL2: B-cell lymphoma-2
bFGF: Basic FGF
BMI: Body mass index
c-MYC: MYC proto-oncogene
COL4A5: Collagen type IV alpha 5
COL4A6: Collagen type IV alpha 6
COL-I: Collagen I
CRC: Colorectal cancer
DNMT: DNA methyltransferase
ECM: Extracellular matrix
FBS: Fetal bovine serum
FGF: Acidic fibroblast growth factor
FH: Fumarate hydratase
GAPDH: Glyceraldehyde-3-Phosphate Dehydrogenase
HMGA2: High mobility group AT-hook 2
HULP: Human uterine leiomyoma primary
MED12: Mediator complex subunit 12
MM: Adjacent myometrium
MMP7: Matrix metalloproteinase 7
PAI-I: Plasminogen activator inhibitor-1
PCNA: Proliferating cell nuclear antigen
RIPA: Radioimmunoprecipitation assay
TGF-β: Tumor growth factor beta
UL: Uterine leiomyoma
UPA: Ulipristal acetate
VEGF: Vascular endothelial growth factor
WB: Western blot
WISP1: Wnt1-inducible-signaling pathway protein 1
