# Supplementary material for: 5-aza-2′-deoxycitidine inhibits cell proliferation, extracellular matrix formation and Wnt/β-catenin pathway in human uterine leiomyomas
Source: Reprod Biol Endocrinol. 2021 Jul 8;19:106. doi: 10.1186/s12958-021-00790-5 (PMC8265104; doi:10.1186/s12958-021-00790-5)
Supplement: Supplementary file 1 — Additional file 1. [file 12958_2021_790_MOESM1_ESM.docx]

**Supplementary Table 1. Clinical variables of patients.**

| PATIENT | AGE | BMI | NUMBER OF LEIOMYOMAS | SIZE OF THE BIGGEST LEIOMYOMA (cm) |
| --- | --- | --- | --- | --- |
| 1 | 45 | 26 | 1 | 10 |
| 2 | 31 | 20 | 1 | 9 |
| 3 | 41 | 23 | 5 | 14 |
| 4 | 39 | 25 | 2 | 18 |
| 5 | 43 | 22 | 2 | 8 |
| 6 | 40 | 29 | 1 | 8 |
| 7 | 46 | 33 | 1 | 12 |
| 8 | 44 | 19 | 4 | 8 |
| 9 | 43 | - | 1 | 8 |
| 10 | 38 | 27 | 1 | 13 |
| 11 | 41 | 20 | 2 | 12 |
| 12 | 48 | 28 | 1 | 13 |
| 13 | 39 | 24 | 7 | 14 |
| 14 | 44 | - | 6 | 9 |
| 15 | 37 | 26 | 4 | 8 |
| 16 | 43 | 23 | 4 | 2 |
| *MEAN* | *41.37* | *24.64* | *2.68* | *9.5* |
| *SD* | *4.09* | *3.89* | *2.02* | *0.70* |
