# Supplementary material for: 5-aza-2′-deoxycitidine inhibits cell proliferation, extracellular matrix formation and Wnt/β-catenin pathway in human uterine leiomyomas
Source: Reprod Biol Endocrinol. 2021 Jul 8;19:106. doi: 10.1186/s12958-021-00790-5 (PMC8265104; doi:10.1186/s12958-021-00790-5)
Supplement: Supplementary file 2 — Additional file 2. [file 12958_2021_790_MOESM2_ESM.docx]

**Supplementary Table 2.**  **Primers sequences**

| GENE | Forward sequence | Reverse sequence |
| --- | --- | --- |
| DNMT1 | 5’-ACG ACC CTG ACC TCA AAT A-3’ | 5’-CAG ACT CGT TGG CAT CAA A-3’ |
| CMYC | 5’-CTGAGGAGGAACAAGAAGATG-3’ | 5’-TGCGTAGTTGTGCTGATG-3’ |
| MMP7 | 5’-GAT GGT AGC AGT CTA GGG ATT A-3’ | 5’-CTG CAT TAG GAT CAG AGG ATT G-3’ |
| GAPDH | 5’- AGATCAAGAAGGTGGTGAAG-3’ | 5’-TTGTCATACCAGGAAATGAGC-3’ |
